# Redox Homeostasis in Ocular Tissues: Circadian Regulation of Glutathione in the Lens?

**DOI:** 10.3390/antiox11081516

**Published:** 2022-08-03

**Authors:** Julie C. Lim, Haruna Suzuki-Kerr, Tai X. Nguyen, Christopher J. J. Lim, Raewyn C. Poulsen

**Affiliations:** 1 Department of Physiology, University of Auckland, Auckland 1023, New Zealand; h.suzuki-kerr@auckland.ac.nz (H.S.-K.); tai.nguyen@auckland.ac.nz (T.X.N.); clim865@aucklanduni.ac.nz (C.J.J.L.); 2 New Zealand National Eye Centre, University of Auckland, Auckland 1023, New Zealand; 3 School of Medical Sciences, University of Auckland, Auckland 1023, New Zealand; r.poulsen@auckland.ac.nz; 4 Department of Pharmacology, University of Auckland, Auckland 1023, New Zealand

**Keywords:** lens, glutathione, circadian rhythms, cataract

## Abstract

Accumulating evidence in tissues suggests an interconnection between circadian clocks and redox regulation. Diurnal variations in antioxidant levels, circadian rhythms of antioxidant enzyme activity, and differences in oxidative stress markers at different times of the day all indicate that oxidative stress responses follow a circadian rhythm. Disruptions of circadian rhythms are linked to a number of age-related diseases, including those in the eye. Typically, ocular tissues contain a robust antioxidant defence system to maintain redox balance and minimise oxidative stress and damage. The lens, in particular, contains remarkably high levels of the antioxidant glutathione (GSH). However, with advancing age, GSH levels deplete, initiating a chain of biochemical events that ultimately result in protein aggregation, light scattering, and age-related cataracts. While there is evidence that the lens exhibits circadian rhythms in the synthesis and release of melatonin, little is known about the regulation or function of timekeeping mechanisms in the lens. Since circadian rhythms are disrupted with age, and the depletion of GSH in the lens is a known initiating factor in the development of age-related cataracts, understanding the mechanisms involved in regulating GSH levels may lead to the future development of approaches to manipulate the clock to restore GSH levels and redox balance in the lens, and protect the lens from cataracts.

## 1. Introduction

Lens cataract is the leading cause of visual impairment and blindness worldwide. With an ageing and diabetic population, the number of individuals with cataracts is increasing, with the current estimate that cataracts afflict more than 20 million people worldwide. Currently, the only way to treat cataracts is by cataract surgery, in which the opaque lens is replaced with an artificial plastic lens. While this effectively restores sight, the demand for cataract surgery far exceeds limited public health resources, and the anticipated economic and social costs of lens cataracts are quite staggering. The alternative approach is to delay the onset of cataracts, which would greatly reduce the need for and expense associated with surgical intervention. In order to achieve this, a better understanding of the molecular, physiological and biochemical changes that occur in the lens with ageing is required. 

The circadian clock is a powerful endogenous cellular timekeeping mechanism that allows organisms to fine-tune their physiology and behaviours to specific times of day [1]. However, our modern 24/7 society produces numerous examples where our lifestyle is in conflict with our internal biological clocks. As a result, there is growing concern regarding the consequences of circadian disruption on human health [2], including age-related eye diseases [3,4] such as cataracts. In the young lens, the high levels of the antioxidant glutathione (GSH) ensure the lens remains clear and transparent [5] so that light rays can be properly focused onto the retina. However, with advancing age, GSH levels are known to decrease specifically in the lens nucleus (centre), resulting in the extensive loss of protein sulfhydryl groups, an increase in protein-thiol mixed disulfides, and an increase in the water-insoluble fraction. This leads to the formation of protein-protein disulfides (PSSP) and other cross-linkages, resulting in protein aggregation and light scattering. This series of biochemical changes has been extensively reviewed [5,6,7,8,9], and there is consensus that oxidative stress is the major contributing factor to age-related nuclear cataract formation. However, despite this, oral administration of nutrients and antioxidants to date has proven to be largely ineffective [10], predominantly due to the difficulty of delivering antioxidants to the lens nucleus at sufficient doses for therapeutic benefit [11]. Therefore, a deeper understanding of the delivery, uptake, metabolism, and regulation of GSH in the lens is required in order to develop approaches that can restore and/or elevate GSH levels, particularly in the lens nucleus and delay or prevent age-related cataracts.

Emerging evidence in non-ocular tissues suggests an interconnection between antioxidant homeostasis and the circadian clock with antioxidants and enzymes that protect the cell from oxidative stress exhibiting daily cycles in their expression or activity levels [12,13,14]. This has led to the idea that circadian rhythms may be involved in controlling GSH levels in the lens and that the age-dependent dysfunction of circadian rhythms may contribute to impairment of GSH homeostasis, with the subsequent manifestation of age-related lens cataract.

To investigate this in more detail, this review will first introduce the concept of the circadian clock and the role of circadian rhythms in enabling us to adapt to changes in our environment and anticipate changes. It will then focus on the molecular circadian clock and detail recent findings that demonstrate the lens to contain core circadian clock components. Next, the importance of redox balance in the lens will be discussed, along with evidence from other tissues that suggest that redox balance may be controlled in a circadian manner. The review will then highlight evidence that suggests that the lens is influenced by a circadian clock and that redox balance in the lens may be circadian regulated. 

## 2. The Circadian Clock

**Figure 1:**
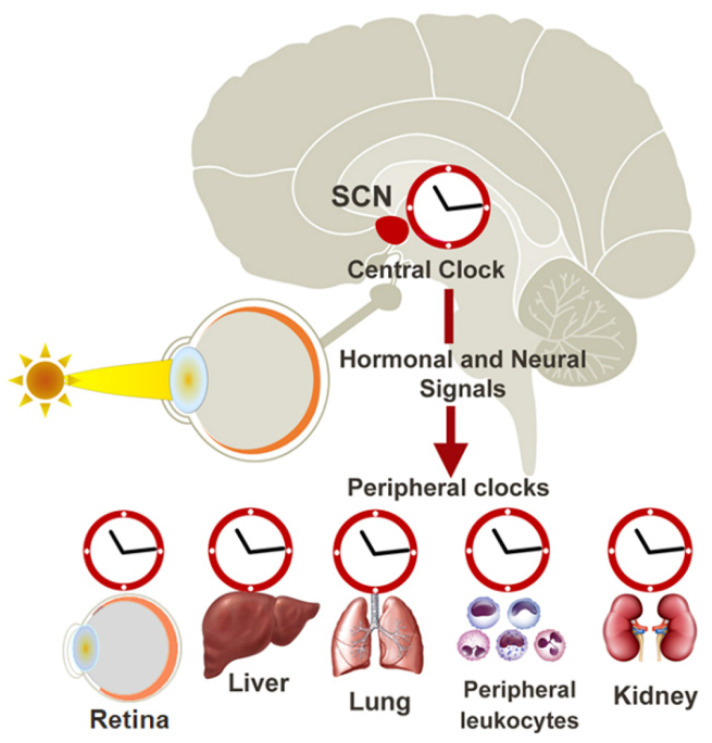
The mammalian circadian clock network. The mammalian circadian clock network consists of the central clock located in the suprachiasmatic nucleus (SCN) of the hypothalamus and peripheral oscillators present in virtually all cell types. Light activates a specific group of photoreceptors in the retina that are connected to the central SCN clock, which synchronises and entrains peripheral circadian clocks via neural and endocrine pathways.

Circadian rhythms are observed in a range of functions in mammals, such as sleep–wake behaviour and body temperature, as well as metabolic, endocrine, cardiovascular, and immune functions [15]. These circadian rhythms are maintained by the “circadian clock” or “biological clock”, which is organised broadly in two parts: (i) the central circadian clock within the brain that governs the circadian rhythms of the whole organism, and (ii) peripheral circadian clocks that operate in a local manner at the tissue level (Figure 1). The central circadian clock is made up of a network of neurons located in the suprachiasmatic nucleus (SCN) of the hypothalamus. It synchronises physiological rhythms with rhythmic changes in the environment. The light-dark cycle is the dominant environmental cue (or zeitgeber) used by humans to entrain to the 24-hour cycle. Photoentrainment is achieved by a class of specialised neurons in the retina called intrinsically photosensitive retinal ganglion cells (ipRGCs), which express melanopsin and are capable of detecting short-wavelengths of light (~480 nm) [16,17]. Light detection by the ipRGCs sends electrical signals to the SCN via the retinohypothalamic tract (RHT), leading to synchronisation of the central circadian clock with the day/night cycle. 

The central circadian clock, in turn, partially regulates circadian clocks in peripheral tissues through the control of factors such as hormones, cytokines, metabolites, sympathetic nervous activation, and body temperature [18]. Controlled rhythmic release of various hormones such as the corticotropin-releasing hormone (CRH), adrenocorticotropic hormone (ACTH) [19], and melatonin [16] influences peripheral circadian clocks via receptors expressed in these tissues, providing a mechanism by which peripheral clocks can also be coordinated to the day/night cycle. Peripheral circadian clocks can also function autonomously from the central clock. Peripheral clocks in the retina, liver, kidney, pancreas, and heart [20] have been shown to continue to function in the absence of any cues from the SCN clock. Interestingly, peripheral circadian clocks can be influenced by cues independent of the central clock, such as feeding patterns, physical activity, and temperature changes, but the precise mechanism is unknown [14]. Peripheral clocks, therefore, play a key role in integrating all the information from the environment to coordinate an appropriate physiological response specific to the organ/tissue. 

## 3. The Molecular Clock

At the molecular level, circadian rhythms are generated by the rhythmic expression of clock genes and the proteins they encode [2,21]. In mammalian cells, the two main molecular mechanisms that drive the circadian expression of clock proteins are the transcriptional-translational feedback loop (TTFL) and oscillations in the post-translational modifications of clock proteins [22]. While the TTFL controls the timing of synthesis of clock proteins, the post-translational modifications control the timing of the activity of those proteins, regulating both sub-cellular trafficking and degradation of the clock proteins [23,24,25,26].

**Figure 2:**
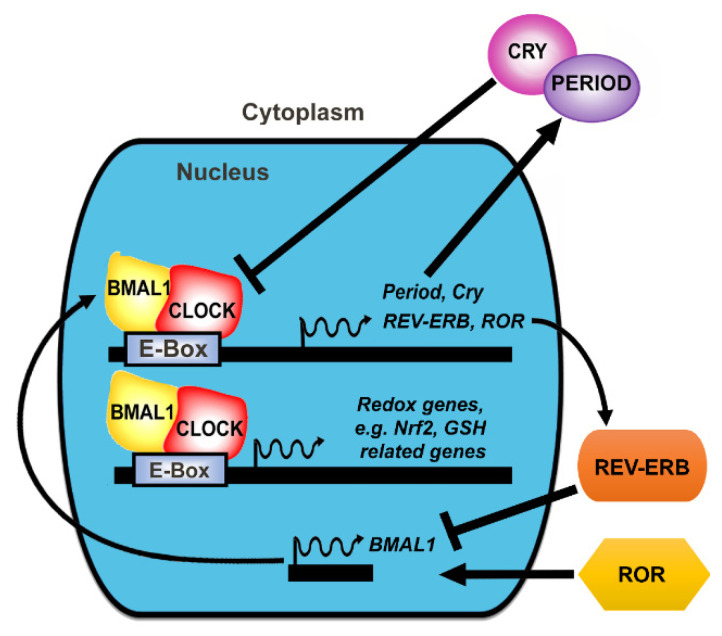
The transcriptional-translational feedback loop. At the molecular level, circadian rhythms are generated by the rhythmic expression of clock genes and the proteins they encode. CLOCK and BMAL1 heterodimers activate transcription of period (Per) and cryptochrome (Cry) genes. PER and CRY proteins, in turn, inhibit their own expression by repressing CLOCK/BMAL1 activity. BMAL1/CLOCK also controls the rhythmic expression of REV-ERBα, which feeds back to suppress BMAL1 transcription. The expression of retinoic acid-related orphan receptor (RORα) is also controlled by BMAL1/CLOCK, which feeds back to activate BMAL1 transcription. BMAL1/CLOCK also activates the transcription of clock-controlled genes such as Nrf2- and GSH-related genes. This feedback loop generates ~24 h oscillations of clock protein levels and activity, which is translated into circadian control of behaviour and physiology.

The TTFL has an interconnected positive limb and a negative limb (Figure 2). The positive limb contains brain and muscle aryl hydrocarbon receptor nuclear translocator (ARNT)-like protein 1 or 2 (BMAL1 or BMAL2), and its dimer partner, circadian locomotor output cycles kaput (CLOCK) protein. The negative limb consists of the period (PER) and cryptochrome (CRY) proteins. Humans express three period isoforms, PER1, PER2, and PER3, and two cytochrome isoforms, CRY1 and CRY2. BMAL1 is a transcription factor that dimerises with CLOCK to directly bind to the E-Box promoter in clock-controlled genes (CCGs). CLOCK also has intrinsic histone acetyltransferase (HAT) activity and acetylates BMAL1 upon dimerisation [27], increasing the binding of the CLOCK/BMAL1 heterodimer complex to the promoter region of the target gene [28], thus regulating transcription. The downstream genes regulated by the CLOCK/BMAL1 heterodimer include genes for core clock components PER and CRY and clock regulators, REV-ERBα and retinoic acid receptor-related orphan receptors (ROR) (see Figure 2) as well as other genes involved in a range of cell processes, including energy metabolism [29], endocrine regulation, immune responses [30] and antioxidant protection [14,31]. CLOCK/BMAL1 activates the transcription of period (Per) and cryptochrome (Cry) genes, and, in turn, PER and CRY proteins inhibit their own expression by repressing CLOCK/BMAL1 activity [32]. This creates an oscillating cycle of expression of BMAL1/CLOCK versus PER/CRY. Auxiliary components of the circadian clock, including nuclear receptors of the REV-ERBα families and ROR families, are also regulated by CLOCK/BMAL1 and, in turn, feedback to regulate the BMAL1/CLOCK-PER/CRY cycle [33,34]. REV-ERBα expression is promoted by BMAL1/CLOCK while suppressed by PER and CRY, and REV-ERBα, in turn, represses BMAL1 and CLOCK expression [34]. ROR competes with REV-ERBα for the BMAL1 promoter, whereas ROR stimulates BMAL1 expression so that the balance of REV-ERBα and ROR proteins influences the level of BMAL1 expression (Figure 2). These feedback loops result in oscillations of clock protein levels and activity over a period of ~24-hrs, which is translated into circadian rhythms in behaviour and physiology. The core circadian clock componentry is conserved across different cell types and tissues [22]. 

## 4. The Lens: Structure and Function

**Figure 3:**
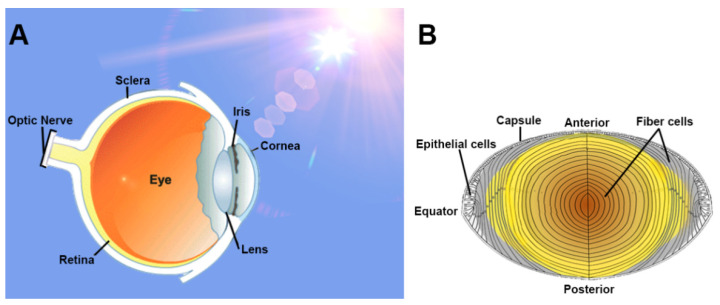
The lens: function and structure. (**A**) Light enters the eye through the transparent cornea and lens, which refracts light to focus onto the retina. Light information is converted into electrical impulses, which are sent to the primary visual cortex of the brain via the optic nerve. The lens is positioned in the anterior region of the eye and is therefore vulnerable to damage from reactive oxygen species (ROS) generated by UV radiation. (**B**) Schematic diagram of the human lens revealing the epithelial monolayer from where epithelial cells proliferate and differentiate at the equatorial zone to form differentiating fibre cells in the outer cortex. Fibre cells progressively lose their organelles, such as mitochondria and nuclei, as they become internalised, creating an inherent age gradient that encapsulates all stages of fibre cell differentiation throughout the lifetime of an individual.

To provide biological context to the influence of a circadian clock in the lens, we have provided a brief overview of the unique structure and function of the lens. The main role of the lens is to focus light onto the retina (Figure 3A). The lens sits suspended within the anterior part of the eye by lens zonules, which enable the lens to change its shape (accommodation) to focus on near or far objects. The anterior surface of the lens is bathed by the aqueous humour, which is secreted by the ciliary epithelium in a circadian manner [35], and in the absence of a blood supply to the lens that would impede the passage of light to the retina, the aqueous humour provides nutrients, amino acids, and antioxidants to the avascular lens. 

Lens transparency is critical for high visual function and is the result of a highly ordered cellular structure [36] (Figure 3B). The lens is enveloped by a capsule, of which beneath the lens anterior surface lies an epithelial cell monolayer. A sub-population of these cells near the lens equator will proliferate, elongate, and differentiate to form differentiating fibre cells [37]. Continuous mitotic division of these cells generates an age gradient across the lens with newly formed and differentiating fibre cells in the periphery or outer cortex [38]. have These cells adopt a flattened hexagonal cross-sectional profile that facilitates their dense packing necessary to preserve transparency. These newly laid down fibre cells slowly displace older fibre cells through the inner cortex towards the centre of the lens (nucleus). As fibre cells are not degraded, older cells remain internalised throughout life, and the lens grows in size and cell number [36]. The ordered fibre cell structure in the deeper lens is not as apparent as in differentiating fibre cells. However, light scattering is eliminated in these deeper regions by a matching of the relative refractive indices between the cytoplasm and membranes of fibre cells [39]. In addition, cells in the deeper lens undergo degradation of light-scattering organelles such as nuclei, mitochondria, and endoplasmic reticulum [40]. The loss of organelles during differentiation means that fibre cells in the nucleus are unable to synthesise proteins de novo which means that this region is particularly susceptible to oxidative damage and cataract formation.

## 5. Evidence of the Molecular Clock in the Lens and Its Importance in Lens Transparency

Recent work by Chhunchha and colleagues [41] identified BMAL1 and CLOCK in the mouse lens and showed BMAL1 and CLOCK to exhibit a rhythmic pattern of expression. The peak expression of clock genes was observed at ZT22-ZT2 (ZT0 was 7 a.m., when lights were turned on), and expression was lowest at ZT6-ZT14. At the protein level, BMAL1 and CLOCK expression also peaked at ZT22 –ZT2, reaching a trough at ZT10-ZT14, revealing that the expression pattern of clock proteins was directly linked to the expression of clock genes. Preliminary work from our laboratory, localising clock genes in the rat lens, has revealed that only the nucleated cells and not the anucleated cells of the lens contain the BMAL1/CLOCK/PER/CRY clock machinery, which is expected since this is dependent on transcription/translation feedback loops. 

BMAL1 and CLOCK have both been shown to be important in maintaining lens transparency [42,43]. BMAL1 KO mice exhibit a significantly reduced lifespan (about 8 months on average) and develop a number of pathologies characteristic of mice with premature ageing phenotypes, including lens cataracts [43]. By 7.5 months, all BMAL1 KO mice (both sexes) developed some form of cataract-either nuclear cataracts, cortical cataracts, or anterior subscapular cataracts in one or both eyes, which were not apparent in age-matched wild-type mice [43]. Histological analysis of the posterior region of the lens of BMAL1 KO mice revealed the presence of infiltrating epithelial cells, which are normally located only in the anterior region of the lens, suggesting that loss of BMAL1 interferes with normal lens development [43]. CLOCK KO mice did not have such a reduced lifespan compared to BMAL1 KO mice but did develop cataracts, although the type of cataract was not specified [42]. By ~28 months, more than 70% of male and 90% of female CLOCK KO mice had cataracts, with cataracts evident as early as 11 months of age for females and 14 months for males [42]. This is a contrast to wild-type mice, where only 30% of mice developed cataracts by ~28 months, and the first appearance of cataracts was detected at ~ 21 months of age for females and 28 months of age for males [42]. Since both BMAL1 and CLOCK form a complex, these findings suggest that BMAL1 and CLOCK play an important role in normal lens physiology. 

## 6. The Importance of Redox Balance in the Lens

**Figure 4:**
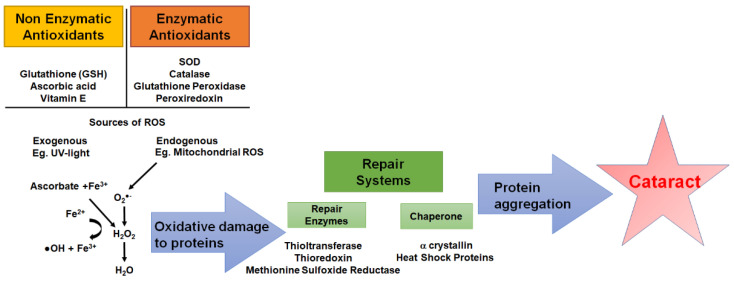
Schematic of the antioxidant defence systems of the lens. To protect against exogenous and endogenous sources of reactive oxygen species (ROS), the lens utilises nonenzymatic antioxidants such as glutathione (GSH), ascorbic acid, and vitamin E along with enzymatic antioxidants such as superoxide dismutase (SOD), which neutralises the superoxide radical (O_2_-•) and catalase, glutathione peroxidase (GPx) and peroxiredoxins, which neutralises H_2_O_2_ into H_2_O and molecular oxygen (O_2_). Repair systems such as the repair enzymes thioltransferase, thioredoxin, methionine sulfoxide reductase, and chaperone proteins work to repair oxidised proteins. However, depletion of antioxidant capacity coupled with a failure to repair damaged proteins results in protein aggregation, increased light scattering, and ultimately cataract formation.

The lens is susceptible to oxidative stress as a result of oxidative insults from exogenous sources (e.g., UV radiation, air pollutants, environmental toxins) and endogenous sources (e.g., cellular metabolism, mitochondrial respiration, inflammation) [44]. To minimise oxidative stress, the lens utilises a range of antioxidant defence systems, which include nonenzymatic and enzymatic antioxidants in combination with repair and chaperone systems [45] (Figure 4). Nonenzymatic antioxidants of the lens include glutathione (GSH), ascorbic acid (or vitamin C), and vitamin E (e.g., *α*-tocopherol). Enzymatic antioxidants include superoxide dismutase (SOD), which catalyses the dismutation of O_2_^•−^ into H_2_O_2_ and O_2,_ and catalase, glutathione peroxidase, and peroxiredoxins, which detoxify H_2_O_2_ to H_2_O. Finally, there are repair (e.g., thioltransferase, thioredoxin, methionine sulfoxide reductase) and chaperone systems (e.g., heat shock proteins, alpha crystallin) which protect proteins under oxidative challenges by dethiolation and/or refolding and stabilising proteins [6]. As we age, our antioxidant defence systems decline, resulting in a depletion of GSH, increased oxidative stress, and ultimately the formation of lens cataracts. Since it is known that circadian rhythms also decline in age, is it possible that this might contribute in part to the decline of the antioxidant defence systems of the lens and the onset of age-related cataracts?

## 7. Evidence of Circadian Regulation of Redox Homeostasis in Non-Ocular Tissues

In humans and animals, many antioxidants that protect the cell from oxidative stress exhibit daily cycles in their expression or activity levels. Presumably, this is to either respond or adapt to the needs of the tissue to keep in line with daily changes in the external and internal environment [13,14,31]. However, emerging evidence suggests that tissues may be able to anticipate times of high ROS exposure through a circadian mechanism to upregulate antioxidants as a pre-emptive approach. 

Jang et al. have reported circadian variations in the expression and activity of SOD1 (Cu/Zn SOD) in mice liver homogenates [46], peaking at ZT9 (9 h after lights-on time) and then decreasing to low levels at ZT21. However, SOD1 rhythmicity was abolished in period2 (Per2) gene KO mice (per2-KO), and the phase of the rhythm was affected in Per1/Per2 double KO (DKO) mice. Moreover, unlike WT mice, Per1/Per2-DKO mice were not able to upregulate SOD1 levels when exposed to the pro-oxidant tert-butylhydroperoxide (t-BHP). These findings suggest that WT mice are able to anticipate and prepare countermeasures for the upcoming active period and accompanying oxidative stresses, whereas mutant mice were more sensitive to oxidative stress due to the disruption of the circadian clock, which, in turn, might disrupt the functioning of antioxidant defence mechanisms in these mice [46].

Oxidative stress biomarkers have also shown circadian rhythmicity in their expression patterns. 8-Oxoguanine DNA glycosylase (OGG1) is a DNA base excision repair pathway involved in the removal of oxidative damage to DNA such as 8-Oxoguanine (8-oxoG). A circadian variation of OGG1 gene expression and activity was found in human lymphocytes with higher levels in the morning compared to the evening hours, and consistent with this, lower levels of 8-oxoG were found in morning hours compared to those in the evening hours [47]. In vitro studies using human dermal fibroblasts revealed that knockdown of BMAL1 resulted in the abolishment of circadian variation of OGG1 expression and an increase in OGG1 activity [47]. These findings provide evidence of circadian regulation of the OGG1 enzyme and that the circadian modulation of the 8-oxoG DNA damage repair system could render humans less susceptible to accumulating 8-oxoG DNA damage in the morning hours.

Previous studies in mammals have reported daily fluctuations in levels of GSH in the liver [48,49], platelets [50], pancreas [51], and cerebral cortex [52]. Enzymes involved in the synthesis of GSH, such as glutamate-cysteine ligase (GCL) and the regeneration of GSH, glutathione reductase (GR), also exhibit rhythms in their activity [49,53,54]. Moreover, NRF2, a transcription factor involved in the transcription of a number of genes involved in antioxidant defence, including glutathione-related enzymes such as GCL, GR, glutathione peroxidase, and glutathione S transferase [55], has been shown to be regulated by a circadian clock [53]. Sun et al. demonstrated that BMAL1 was found to bind to the promoter of the Nrf2 gene through an E-Box element associated with strongly rhythmic activation of NRF2 in rat kidneys [56]. When NRF2 expression was low, the expression of downstream antioxidant proteins such as the modulatory subunit of GCL (GCLM) was also low, and this coincided with more serious renal oxidative stress injury and pathological and functional impairment induced by ischaemia reperfusion [56]. In the rat lung, it has also been shown that the recruitment of NRF2 to gene-specific antioxidant gene promoters that drives the expression of genes involved in GSH synthesis as well as GSH levels is under rhythmic clock regulation [53]. NRF2-deficient mouse embryonic fibroblasts were not capable of maintaining circadian rhythms of a number of key antioxidant genes involved in GSH homeostasis and showed arrhythmic and reduced levels of GSH [53]. Moreover, in the lungs of *ClockΔ19* mutant mice, NRF2 expression and GSH levels were reduced and associated with increased oxidative damage and a spontaneous fibrotic-like pulmonary phenotype [53], highlighting the essential role of Clock/NRF2 in mediating rhythmic GSH levels and protection against oxidative stress.

Taken together, it appears that in other tissues, antioxidant and oxidative stress responses follow a circadian rhythm where there are differences in antioxidants and oxidative stress components at different times of the day. However, what evidence is there that antioxidant defence systems may be regulated in ocular tissues to anticipate the need for ocular tissues to protect against oxidative insults?

## 8. Evidence of Circadian Regulation of Redox Homeostasis in the Lens

It is known that ocular tissues utilise circadian rhythms of activity to regulate important functions such as visual processing [57], neurotransmitter synthesis and release [58], disc shedding and phagocytosis [59,60,61], corneal thickness and intraocular pressure [62,63], but what evidence exists that suggest the lens is influenced by a circadian clock and/or uses a circadian clock to influence the regulation of GSH and/or other redox components in the lens?

The lens contains high concentrations of GSH to enable it to withstand UV light-induced oxidative insults and afford protection from ROS generated as by-products of cellular metabolism. GSH is the principal antioxidant in the lens (exists at concentrations ~4–6 mM in the lens) [5,7] and plays multiple roles in the lens, including detoxification of ROS, protection of sulfhydryl groups from oxidation [64], detoxification of xenobiotic compounds by GSH conjugation [65], and the regeneration of antioxidants such as ascorbic acid [66]. GSH levels in the lens exist as a gradient with levels of GSH highest in the epithelium and outer cortex and lowest in the nucleus. The depletion of GSH is a known initiating factor in the onset of age-related cataract formation [67,68,69]. With increasing age, GSH levels in the lens nucleus deplete, and when levels fall below a concentration of 1 mM, a series of events are initiated [8]. These include extensive oxidation of the -SH groups in proteins [70,71], loss of protein solubility [68], the formation of disulphide bonds leading to protein aggregation [72], and eventually cataract formation. However, maintaining GSH homeostasis is challenging, particularly for an avascular tissue such as the lens, which is solely reliant on the aqueous humour for nutrients, GSH, and the amino acids required for GSH synthesis. The aqueous humour is secreted in a circadian manner, with its secretion higher during the day than at night [73]. This suggests that delivery of GSH and GSH precursor amino acids to the lens is also not constant and that GSH levels in ocular tissues may display circadian behaviour.

This idea is strengthened by evidence that the lens exhibits circadian behaviours in the synthesis of melatonin [74,75,76]. Abe et al. showed that in rabbit lenses, melatonin and serotonin *N*-acetyl-transferase (AANAT) activity, which catalyses the conversion of serotonin to *N*-acetylserotonin and regulates melatonin synthesis, showed significant day and night changes with high levels during the night (24:00–01:00) and low levels during the day (12:00–13:00) [74]. Subsequently, Abe et al. showed that in cultured rat lenses, melatonin synthesis also exhibited a circadian rhythm with AANAT activity lowest during the day and highest during the night [75]. This diurnal pattern persisted in constant darkness, which implied that AANAT activity in cultured rat lenses was not entrained by a light/dark cycle and that circadian rhythms in the lens were independent of the central clock in the SCN [75]. This finding may imply that the lens peripheral clock is controlling melatonin synthesis. More recently, melanopsin photopigment was discovered in human lens epithelial cells [76]. It was postulated that when light activated melanopsin, this led to a decrease in melatonin synthesis. Under constant darkness, it was revealed that the expression of AANAT increased by approximately 2.5 times, leading to increased melatonin production in human lens epithelial cells compared to light conditions. When a selective melanopsin inhibitor AA92593 was applied to cells, AANAT expression and melatonin levels did not change between light and dark conditions [76]. Hence, the change in melatonin levels depended on the activation of melanopsin by light and was linked to changes in the expression of the AANAT enzyme. The control of AANAT expression by the light suggests a regulation comparable to the one driven by SCN but with no connection to the neural component suggesting that the lens may be able to act as a peripheral clock capable of maintaining the same rhythmicity in melatonin synthesis as that of the SCN and pineal gland. In addition, the discovery of melanopsin in the lens opens the possibility of regulating melatonin synthesis through changing illumination conditions and increasing melatonin levels in the lens to protect against oxidative stress. 

While little is known about the regulation or function of timekeeping mechanisms in the lens, BMAL1and CLOCK are known to be expressed in the lens [41]. In addition, Chhunchha et al. revealed NRF2 and one of its target genes, peroxiredoxin 6 (Prx6), which is involved in the detoxification of H_2_O_2_ to exhibit a rhythmic expression that was synchronised with BMAL1 and CLOCK expression in mouse lenses in vivo [41]. Moreover, shRNA targeted knockdown of BMAL1 in human lens epithelial cells disrupted NRF2 expression, as well as expression of Prx6 and other NRF2-regulated genes such as SOD1 and SOD2 [41]. This, in turn, resulted in increased ROS levels, with cells exhibiting an increased sensitivity to oxidative stress-induced cell death [41]. Interestingly ROS levels were reciprocally linked with levels of BMAL1/NRF2/antioxidant genes. These findings suggest that BMAL1 is an essential component for the regulation of NRF2-mediated antioxidant protective response to minimise ROS levels within the lens and protect against oxidative damage. Since NRF2 is known to regulate expression of genes involved in the synthesis and regeneration of GSH, BMAL1/NRF2 may also play a role in the regulation of GSH levels in the lens and, if so, may be targeted for enhancing GSH levels in the lens to delay age-related cataracts.

Taken together, this collective evidence suggests that the lens contains the molecular machinery to be able to regulate GSH levels in a circadian manner. Since circadian rhythms are disrupted with advancing age [77,78], this implies that circadian rhythms may be involved in controlling GSH homeostasis in the lens and that the age-dependent dysfunction of circadian rhythms may contribute to impairment of GSH homeostasis, with the subsequent manifestation of age-related eye diseases such as lens cataracts.

## 9. Conclusions

This review demonstrates that circadian rhythms contribute to the control of redox balance in other tissues. However, future work is required to determine if clock component genes and redox genes in the lens oscillate in a circadian manner to regulate GSH homeostasis. This is important since GSH levels in the lens are known to decline with age, and understanding these mechanisms may provide novel ideas for manipulating the clock to restore redox balance and protect the lens from age-related cataracts.
